# Validation of FRIEND and ACSM Equations for Cardiorespiratory Fitness: Comparison to Direct Measurement in CAD Patients

**DOI:** 10.3390/jcm9061889

**Published:** 2020-06-17

**Authors:** Won Young Jang, Dong Oh Kang, Yoonjee Park, Jieun Lee, Woohyeun Kim, Jah Yeon Choi, Seung-Young Roh, Yuna Jang, Se-Hyun Park, Woo-Sub Kim, Jin Oh Na, Cheol Ung Choi, Seung-Woon Rha, Chang Gyu Park, Hong Seog Seo, Eung Ju Kim

**Affiliations:** 1 Cardiovascular Center, Division of Cardiology, Department of Internal Medicine, Catholic University of Korea St. Vincent Hospital, The Catholic University of Korea College of Medicine, Suwon 16247, Korea; raph83@naver.com; 2 Cardiovascular Center, Division of Cardiology, Department of Internal Medicine, Korea University Guro Hospital, Korea University College of Medicine, Seoul 08308, Korea; gelly9@naver.com (D.O.K.); ch93lje@naver.com (J.L.); coincidence1@naver.com (W.K.); kekeruki@gmail.com (J.Y.C.); rsy008@gmail.com (S.-Y.R.); liastorm@naver.com (Y.J.); koolup93@gmail.com (J.O.N.); wmagpie@korea.ac.kr (C.U.C.); swrha617@yahoo.co.kr (S.-W.R.); parkcg@kumc.or.kr (C.G.P.); mdhsseo@unitel.co.kr (H.S.S.); 3 Division of Cardiology, Department of Internal Medicine, Heart Vascular Stroke Institute, Samsung Medical Center, Sungkyunkwan University School of Medicine, Seoul 06351, Korea; yoonjeedrpark@gmail.com; 4 Sport Science Center, Korea University Guro Hospital, Seoul 08308, Korea; 57270115@naver.com; 5 Department of rehabilitation, Korea University Guro Hospital, Korea University College of Medicine, Seoul 08308, Korea; jelmanoo@naver.com

**Keywords:** cardiorespiratory fitness, validation studies, coronary artery disease

## Abstract

The regression equation of the American College of Sports Medicine (ACSM) was a preferred method for estimating maximal oxygen consumption (VO2max). Recently, a more precise equation from the fitness registry and the importance of exercise national database (FRIEND) for healthy people was developed. This study compared VO2max estimated by the ACSM and FRIEND equations to VO2max directly measured in coronary artery disease (CAD) patients. Overall, 293 CAD patients who participated in cardiac rehabilitation between June 2015 and December 2018 were analyzed. Directly measured VO2max values were compared to the ACSM and FRIEND equations. The directly measured VO2max was significantly different from the estimated VO2max by ACSM equation (24.16 vs. 28.7 mL/kg/min, *p* < 0.001), which was overestimated by 20% in men and 16% in women. However, there was no statistically significant difference between the directly measured VO2max and the estimated VO2max by the FRIEND equation. (24.16 vs. 24.15 mL/kg/min, *p* = 0.986). In CAD patients, the estimated VO2max from the ACSM equation was significantly higher than the directly measured VO2max. In addition, estimated cardiorespiratory fitness (CRF) by the FRIEND equation showed similar results with directly measured CRF. As a result, the FRIEND equation can predict CRF more accurately than the ACSM.

## 1. Introduction

Cardiorespiratory fitness (CRF) is defined as the circulatory and respiratory ability that supplies oxygen properly to the skeletal muscles during physical activity and the ability of the muscle to extract oxygen [1]. Many studies have shown that a better CRF lowers the risk of development and recurrence of coronary artery disease (CAD) and all-cause mortality [2,3,4,5,6]. Performing exercise tests for CRF measurement is strongly recommended in CAD patients without any contraindications [7]. CRF is measured by maximal oxygen uptake (VO2max). This metabolic demand for a given work rate has been expressed in metabolic equivalents (METs). One MET is equivalent to the amount of oxygen consumed at rest (~3.5 mL O2/kg/min) [8,9].

The direct measurement of VO2max is generally used in research or clinical settings. VO2max is measured when an individual’s physiological limits are reached. Historically, the achievement of VO2max has been defined by the peak of VO2 between the final two exercise work rates, indicating that maximum effort is achieved and maintained for a specified period [7]. The determination of this period can be difficult to define, because it is subjective and rarely observed in exercise tests of patients with cardiovascular or pulmonary diseases [7]. Therefore, various criteria are used to assess maximal efforts, such as a VO2 plateau, blood lactate accumulation, maximal heart rate (HR), rating of perceived exertion and elevated respiratory exchange ratio (RER) ≥ 1.0, 1.10, or 1.15 [10,11,12,13,14,15]. In the absence of electrocardiographic or hemodynamic abnormalities, generally RER ≥ 1.10 indicates an excellent effort and ≥ 1.00 indicates an acceptable effort [11].

The measurement of VO2max requires a special instrument such as a gas analyzer. When the direct measurement of VO2max is not feasible to estimate VO2max, a variety of submaximal and maximal exercise tests can be used [9]. The regression equation was developed to estimate VO2max without a gas analyzer. The generally used regression equation is the American College of Sports Medicine (ACSM) equation (Table 1). This equation was developed nearly four decades ago, and it is well-known for overestimating VO2max when the exercise protocol is too aggressive for a given individual, or when an individual heavily relies on handrail support during the exercise test [7]. The ACSM equation generally estimates a VO2max of 1–1.5 MET higher [16,17,18,19].

Recently, a new regression equation from the fitness registry and the importance of exercise national database (FRIEND), called the FRIEND equation (Table 1), was reported to accurately estimate peak MET levels. The FRIEND cohort, established in 2014, included 7983 healthy adults for discovering normative CRF values in the United States [16]. The FRIEND equation estimates VO2max more accurately than the traditional ACSM equation, with an overall error four times lower than the ACSM equation [20]. The researchers suggested that the potential limitation of the FRIEND equation is the exclusion of diseased populations in the FRIEND registry [20]. In this study, we confirmed the accuracy of both equations by comparing the estimated VO2max from the ACSM and FRIEND equations, to directly measured VO2max in CAD patients. 

## 2. Methods

This cross-sectional study measures the CRF of CAD patients. We analyzed 293 CAD patients who underwent percutaneous coronary intervention (PCI) and who participated in cardiac rehabilitation (CR), between May 2016 and December 2018. Exclusion criteria were pulmonary disease, chronic kidney disease on hemodialysis, malignancy within 5 years and orthopedic injuries.

The patients underwent a treadmill test utilizing the Bruce Ramp protocol that was known to be safe for patients with CAD and measure exercise capacity more accurately [9,21,22,23]. The treadmill test was performed within 1~2 weeks after PCI at Korea University Guro hospital. Before test, patients’ blood pressure, HR and echocardiography were evaluated for risk assessments. The direct gas analysis was performed by Quark (COSMED, Rome, Italy) during an exercise test under 12-lead electrocardiography monitoring, blood pressure and HR measurement, at every stage. Speed and grade were increased every 40 s. The termination criteria were patient request, ventricular arrhythmia, ST-segment depression over 1mm on electrocardiography monitoring, blood pressure drop over 10mmHg, and patient complaint chest discomfort. The peak RER, exercise duration, VO2max, and METs were measured. VO2 values were determined every 40 s stages. VO2max was defined as the highest VO2 value during the exercise phase. The workload in the completed last stage during exercise phase was used for estimation in most subjects. However, near-completed last stage during exercise phase was sometimes used for estimation. The stage is selected according to the higher VO2 value. Directly measured VO2max values were compared to the estimated VO2max by the ACSM and FRIEND equations.

### Statistical Analysis

Continuous data are presented as mean ± standard deviation (SD), whereas categorical data are presented as frequencies (percentages). The student’s t-test and chi-square analysis were used to assess the differences between the sexes. The difference between directly measured CRF and estimated CRF was analyzed by a *t*-test. IBM SPSS 20.0 (Chicago, IL, USA) was used for analysis. Microsoft Excel 2016 (Redmond, WA, USA) was used for equivalence analyses, such as mean bias error (MBE), mean square error (MSE), mean absolute percent error (MAPE), Lin’s concordance correlation coefficient (CCC), scatter plot and Bland–Altman plot. All registry data were blindly reviewed by a physical therapist and a cardiologist. The walking ACSM equation for estimating VO2max is used for all participants, because the highest speed of the participants is 4.1mph.

This study was approved by the Institutional Review Board (IRB) of Korea University Guro Hospital (IRB number 2020GR0060). The requirement for written informed consent was waived because of the retrospective design of the study.

## 3. Results

### 3.1. Baseline Characteristics

The mean age of subjects was 60.7 ± 9.8 years. Most of the participants were men (78%, *n* = 229). The diagnoses of the participants were 64% angina and 36% myocardial infarction (MI). A total of 246 participants (84%) showed a normal systolic function on echocardiography. Only 46% of the participants showed RER ≥ 1.1 (excellent effort) and 81% of the participants showed RER ≥ 1.0 (acceptable effort and excellent effort) during the exercise test. The remaining 19% of participants showed RER < 1.0 (submaximal effort). There was no sex difference in acceptable effort. However, there was a significant difference in excellent effort among sexes; 51% was achieved by men and only 28% was achieved by women (Table 2).

### 3.2. Comparisons of CRF between Direct Methods and Estimation Methods by Two Regression Equations

**Figure 1:**
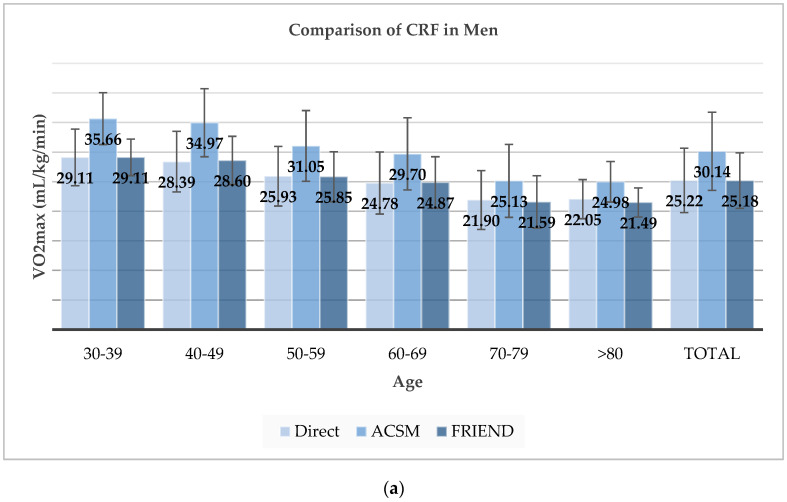

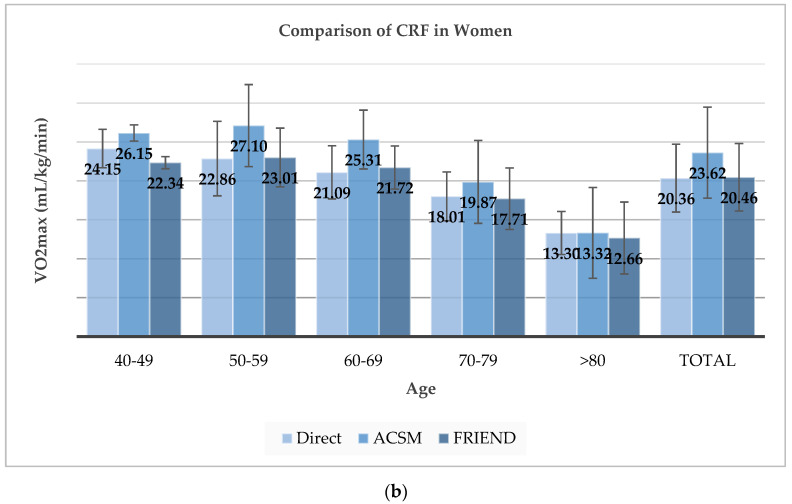
Comparison of cardiorespiratory fitness (CRF) between direct measured CRF and estimated CRF by American College of Sports Medicine (ACSM) and fitness registry and the importance of exercise national database (FRIEND) equation: (**a**) Comparison of CRF in men; (**b**) Comparison of CRF in women.

In all participants, the directly measured VO2max was significantly different from the estimated VO2max by ACSM equation (24.16 ± 5.59 vs. 28.72 ± 7.00 mL/kg/min, *p* < 0.001). However, there was no statistically significant difference in the directly measured VO2max and the estimated VO2max by FRIEND equation (24.16 ± 5.59 vs. 24.15 ± 5.02 mL/kg/min, *p* = 0.986). Compared to the directly measured VO2max, the ACSM equation overestimated VO2max by 19% (20% in men and 16% in women) and the FRIEND method showed a difference < 1% in both sexes. The comparison results were similar across all age groups. Thus, regardless of sex and age, the FRIEND equation could estimate CRF more accurately than the ACSM equation (Figure 1, Table 3).

### 3.3. Bland–Altman Plot for Comparing Directly Measured and Estimated CRF

**Figure 2:**
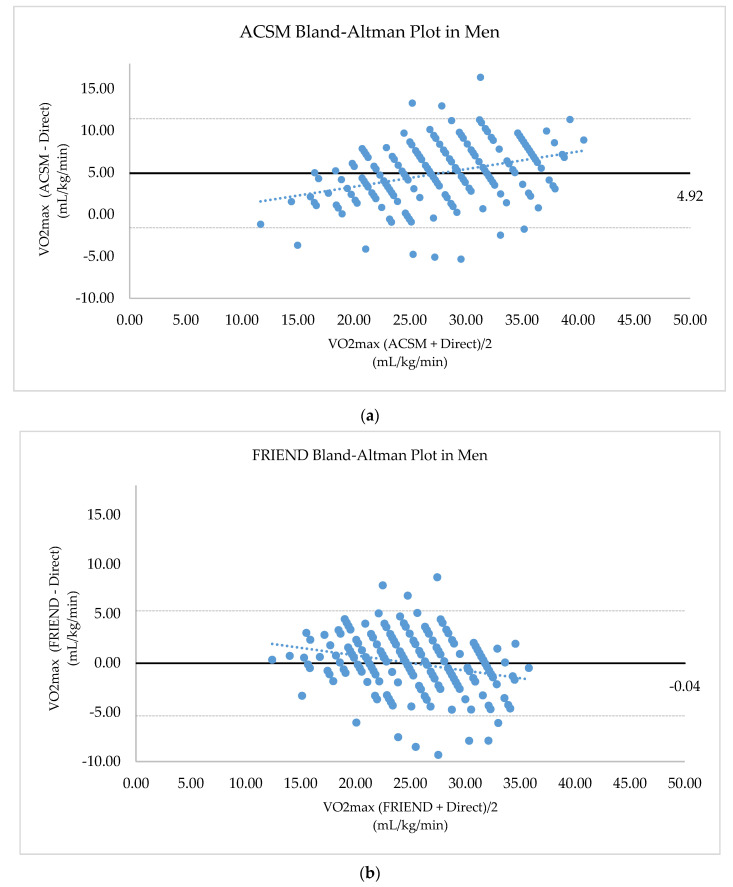

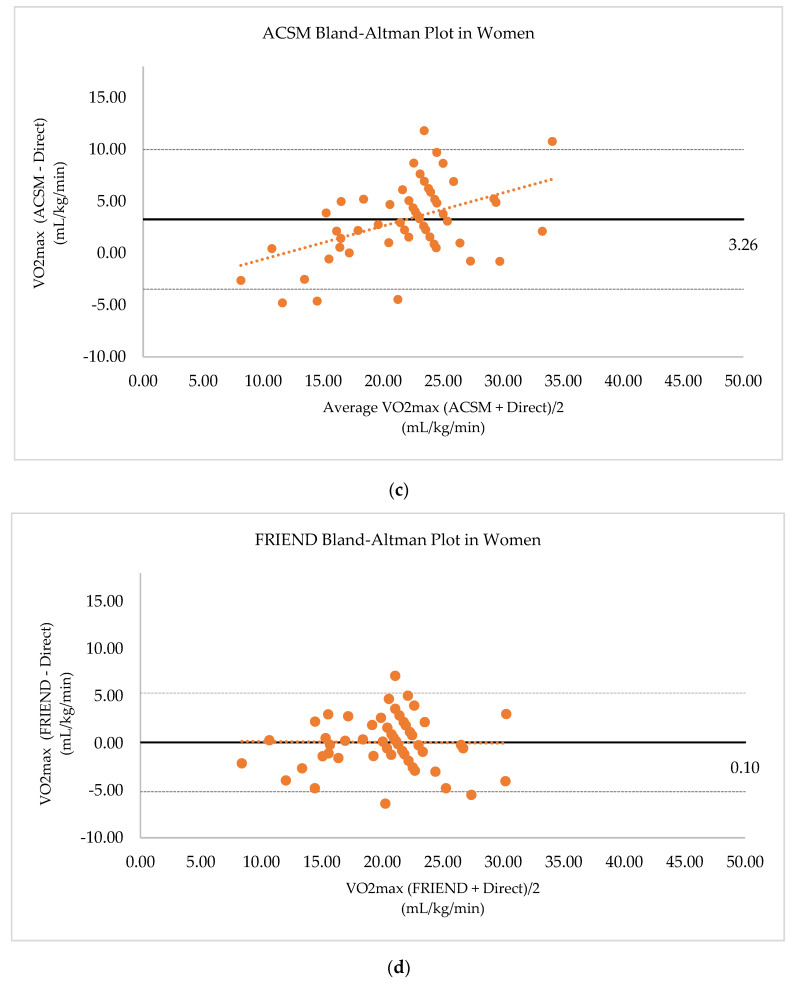
Bland–Altman plot between directly measured CRF and estimated CRF: (**a**) Bland–Altman plot between directly measured CRF and estimated CRF by ACSM equation in men; (**b**) Bland–Altman plot between directly measured CRF and estimated CRF by FRIEND equation in men; (**c**) Bland–Altman plot between directly measured CRF and estimated CRF by ACSM equation in women; (**d**) Bland–Altman plot between directly measured CRF and estimated CRF by FRIEND equation in women. dotted line; linear regression line, horizontal to X-axis linear line; mean difference value, horizontal to X-axis dotted line; upper and lower limits of agreement.

The ACSM equation tends to overestimate VO2max more when higher VO2max. Unlike the ACSM, the FRIEND equation estimates VO2max slightly higher when VO2max is low; the difference is decreased as CRF becomes higher. The FRIEND equation estimates VO2max slightly lower when the VO2max is high. The ACSM equation estimated VO2max, with a mean difference of 4.92 mL/kg/min, higher and the FRIEND equation estimated VO2max, with a mean difference of 0.04 mL/kg/min, lower than VO2max by the direct measurement. (Figure 2).

### 3.4. Equivalence Analysis of the ACSM and FRIEND Equations

**Figure 3:**
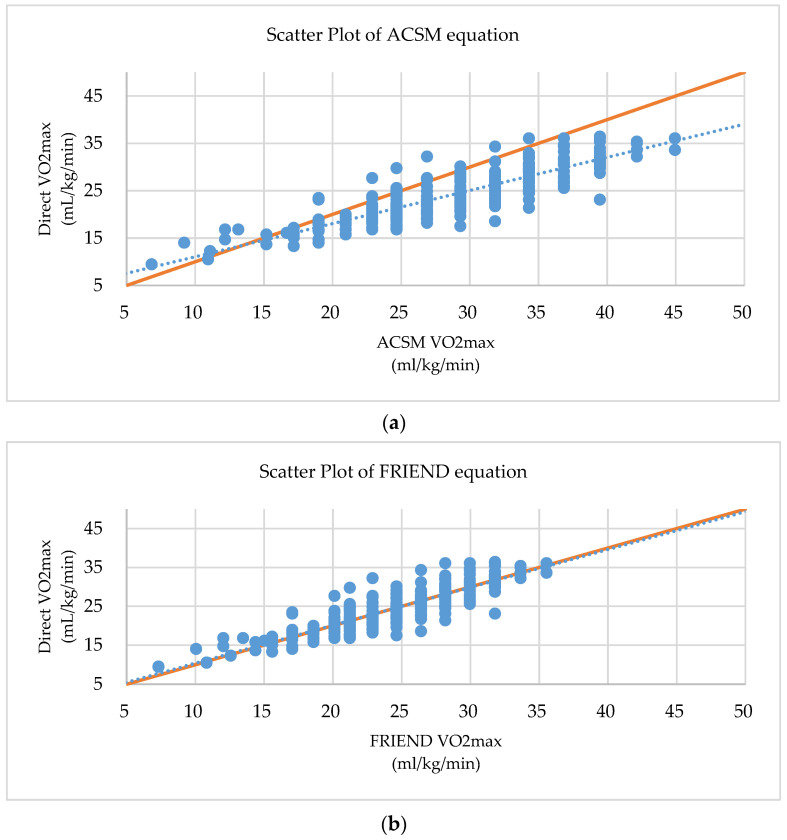
Scatter Plot between directly measured CRF and estimated CRF: (**a**) Scatter plot between directly measured CRF and estimated CRF by ACSM (**b**) Scatter plot between directly measured CRF and estimated CRF by FRIEND equation. Dotted line; linear regression line, Solid line; 1:1 reference line.

For the equivalence analysis of the two equations, MBE, MSE, MAPE, Lin’s CCC and scatter plot were calculated (Table 4, Figure 3). The MAPE value is the commonly used statistical method for equivalence of predictive test, and the Lin’s CCC value is the golden standard statistical method for the equivalence comparison of the new test and standard test. The MAPE value of the ACSM equation is 20.963, and the one of the FRIEND equation is 8.937. The Lin’s CCC value of the ACSM equation is 0.678, and one of the FRIEND equations is 0.870 (Table 4, Figure 3). These results were similar regardless of taking medication or clinical presentation (Tables S1 and S2 in the supplement).

### 3.5. CRF Difference According to Maximal Effort

RER is an indicator of maximal effort. A RER ≥ 1.1 is defined as an excellent effort, a RER ≥ 1.0 is defined as an acceptable effort, and a RER < 1.0 is considered as a submaximal effort. VO2max value was decreased when participants did a submaximal effort, in all measurement methods (Table S3 in the supplement).

## 4. Discussion

The importance of CRF continues to grow, another so-called vital sign. CRF measurement is important, not only in healthy adults, but also in CAD patients for secondary prevention [6,24]. CRF in CAD patients is significantly decreased compared to that in healthy people. The decrease of CRF is one of the strong risk factors for mortality compared to a traditional risk factor such as hypertension, dyslipidemia, smoking, alcohol consumption and diabetes [25,26]. For this reason, exercise-based CR is strongly recommended for CAD patients to achieve healthy lifestyle and risk factor management [27]. The ultimate objective of CR is to improve patients’ life quality by restoring CRF [28]. In addition, the improvement of CRF is associated with a significant reduction of future cardiovascular fatal and nonfatal events, independent of other risk factors [29]. Therefore, the exercise test in CAD patients referred for CR is essential for a baseline assessment of CRF, assessment of prognosis, and the evaluation of the results of training [7].

The direct measurement of the VO2max, which is a golden standard method for assessing CRF, requires a machine capable of measuring gas exchange analysis, which makes it difficult to perform in all institutions. In the absence of such equipment, the CRF is estimated by an equation. The widely used equation for estimating CRF through treadmill test is the ACSM equation, which was published four decades ago [30]. In addition, it is well known that the ACSM equation overestimates CRF compared to the actual CRF [16,17,18]. Recently, the FRIEND equation for healthy adults in the United States was developed with more accuracy [20]. The difference between the FRIEND equations according to the various treadmill protocols was also reported. In the case of using the Bruce ramp protocol, the FRIEND equation overestimated CRF by 12.8% on average and the ACSM equation by 39.8%, on average [20].

The Bruce ramp protocol is known to be safe and widely used for CAD patients [21]. In this study, we analyzed the results of the Bruce ramp protocol in CAD patients. Interestingly, the FRIEND equation accurately estimated CRF within a 1% error for both men and women, whereas the ACSM equation overestimated CRF by 20% in men and by 16% in women. In the results of the equivalence analysis, the MAPE value of the ACSM equation is higher than the one of the FRIEND equation (20.96% vs. 8.94%). MAPE is a most commonly used statistical measure for predictive accuracy of a predictive method. The values of a MAPE, <10, 10~20, 20~50 and >50, are assessed as a highly accurate, a good, a reasonable and an inaccurate prediction, respectively. In addition, the Lin’s CCC value of the FRIEND equation is higher than the value of the ACSM equation (0.870 vs. 0.678). The Lin’s CCC is the concordance between a testing and a gold standard testing. This statistic quantified the agreement between these two measurements. Historically, a Lin’s CCC value greater than 0.80 was an excellent strength of agreement, which means that a new test can be used, instead of the golden standard test. However, McBride recommends more strict requirements for Lin’s CCC value that 0.90~0.95, 0.95~0.99 and greater than 0.99 are assessed as a moderate, a substantial and an excellent strength of agreement. As a result of an equivalence analysis in this study, the newly reported FRIEND equation showed better results in the MAPE and the Lin’s CCC values than the currently widely used ACSM equation.

The results suggest that a considerably accurate CRF can be estimated by the FRIEND equation in CAD patients, performing the treadmill test with the Bruce ramp protocol. It could be very useful in environments where CRF cannot be directly measured in CAD patients, regardless of age and sex.

The RER is used as an indicator of whether the exercise test is enough for a maximal effort. Only 46% of the participants showed RER ≥ 1.1, which means excellent effort, and 81% of the participants showed RER ≥ 1.0, which means acceptable effort during the exercise test. An RER ≥ 1.1 during the treadmill test might not be easy for CAD patients, especially women (only achieved by 28% of participants). Interestingly, the ACSM equation significantly overestimated the CRF more when the CRF was obtained with acceptable effort (RER ≥ 1.0), compared to the directly measured CRF. In cases of submaximal effort (RER < 1.0), the ACSM equation did not significantly overestimate CRF. On the contrary, the FRIEND equation estimated CRF close to the directly measured CRF, regardless of RER. In addition, CRF estimated by the FRIEND equation did not show a statistically significant difference from the directly measured CRF. In other words, unlike the ACSM equation, the FRIEND equation can estimate VO2max accurately for CAD patients, regardless of RER.

The strength of this study is that the FRIEND equation developed for healthy population was first validated to accurately estimate the VO2max in CAD patients. Secondly, this is the first external validation of the FRIEND equation. Despite the possibility of inter-ethnic difference, the equation showed considerable accuracy to estimate CRF in Korean CAD patients. Third, the Bruce ramp protocol, which showed the biggest difference among exercise protocols in CRF estimation in healthy people in the FRIEND study, can be applied for CAD patients with more accurate VO2max estimation by the FRIEND equation. However, further study is needed to determine whether this difference is due to inter-ethnic difference or difference between the healthy population and the CAD population.

The limitations of this study are a cross-sectional registry study conducted at a single center. Second, participants in the study were those who participated in CR after PCI and may be limited to represent all CAD patients. Third, the usefulness of the FRIEND equation for CRF estimation in patients with other diseases requires further study. Fourth, patients with a low CRF who found it difficult to perform the treadmill tests participated less. Lastly, this study was conducted with a relatively small number of participants, especially with fewer women among all participants.

## 5. Conclusions

In CAD patients, the estimated CRF by the ACSM equation was overestimated by 20% in men and 16% in women. Additionally, estimated CRF by the FRIEND equation showed similar results with the directly measured CRF. As a result, the FRIEND equation can estimate CRF more accurately than the ACSM equation. Interestingly, even now, the treadmill machines in most laboratories still estimate MET using the ACSM equation, which is over 40 years old. It is time to update this equation.

## Figures and Tables

**Table 1:** Two regression equations for estimating cardiorespiratory fitness (CRF) with treadmill parameters.

ACSM Equation; Most Widely Used Regression Equation
Running: VO2max = (0.2 × speed) + (0.9 × speed × fractional grade%) + 3.5
Walking: VO2max = (0.1 × speed) + (1.8 × speed × fractional grade%) + 3.5
**FRIEND Equation; Newly Reported Regression Equation**
VO2max = speed × (0.17 + fractional grade × 0.79) + 3.5

VO2max; (mL/kg/min).

**Table 2:** Baseline characteristics of the coronary artery disease patients.

	Total	Men	Women	*p*-Value
Sample size, number	293 (100)	229 (78)	64 (22)	
Age, years	60.7 ± 9.8	59.5 ± 9.8	65.0 ± 8.8	<0.001
Height, cm	165.6 ± 9.1	169.0 ± 6.4	153.4 ± 6.2	<0.001
Weight, kg	69.6 ± 11.8	72.3 ± 10.8	59.8 ± 9.6	<0.001
BMI, kg/m^2^	25.3 ± 3.0	25.3 ± 2.9	25.3 ± 3.2	0.881
Waist to hip, ratio	0.90 ± 0.06	0.90 ± 0.06	0.90 ± 0.06	0.776
Basal metabolic rate, kcal/day	1457 ± 220	1537 ± 188	1198 ± 122	<0.001
Resting SBP, mmHg	121.7 ± 15.9	121.3 ± 15.6	123.4 ± 16.7	0.354
Resting DBP, mmHg	73.8 ± 11.7	73.9 ± 11.4	73.2 ± 12.4	0.674
Heart rate, bpm	79.8 ± 12.3	79.8 ±12.39	79.7 ± 12.2	0.944
Hemoglobin, g/dL	13.6 ± 1.4	13.9 ± 1.3	12.5 ± 1.3	<0.001
Muscle mass, kg	27.8 ± 5.6	30.0 ± 4.2	20.6 ± 3.4	<0.001
Ejection fraction, %	60.3 ± 7.7	60.0 ± 7.7	61.4 ± 7.4	0.171
Normal systolic function, number	246 (84)	190 (83)	56 (88)	0.383
PASP, mmHg	26.5 ± 5.4	26.5 ± 5.1	26.5 ± 6.5	0.980
E/e’, ratio	10.69 ± 3.46	10.32 ± 3.20	11.98 ± 4.02	0.003
Underlying disease				
Hypertension	174 (59)	133 (58)	41 (64)	0.389
Diabetes mellitus	97 (33)	78 (34)	19 (30)	0.511
Hyperlipidemia	147 (50)	112 (49)	35 (55)	0.414
Medication				
Beta blocker	159 (54)	127 (55)	32 (50)	0.438
Calcium channel blocker	86 (29)	65 (28)	21 (33)	0.492
Diagnosis				
STEMI	57 (19)	51 (22)	6 (9)	0.021
NSTEMI	49 (17)	43 (19)	6 (9)	0.075
Unstable angina	119 (41)	83 (36)	36 (56)	0.004
Stable angina	68 (23)	52 (23)	16 (25)	0.701
RER, ratio		1.10 ± 0.12	1.04 ± 0.13	<0.001
RER ≥ 1.0	236 (81)	186 (81)	50 (78)	0.560
RER ≥ 1.10	134 (46)	116 (51)	18 (28)	0.001

Data are presented as mean ± standard deviation or as numbers (percentages of total). BMI; body mass index, SBP; systolic blood pressure, DBP; diastolic blood pressure, PASP; pulmonary artery systolic pressure, STEMI; ST elevation myocardial infarction, NSTEMI; non-ST elevation myocardial infarction, RER; respiratory exchange ratio.

**Table 3:** Comparison of CRF between directly measured CRF and estimated CRF, by ACSM equation and FRIEND equation.

VO2max ± SD (mL/kg/min)								
	N	Direct	ACSM	Ratio	*p*-Value	FRIEND	Ratio	*p*-Value
Total	293	24.16 ± 5.59	28.72 ± 7.00	1.19 ± 0.15	<0.001	24.15 ± 5.02	1.01 ± 0.12	0.986
**Men**	**VO2max** **± SD (mL/kg/min)**							
**Age**	**N**	**Direct**	**ACSM**	**Ratio**	***p*-Value**	**FRIEND**	**Ratio**	***p*-Value**
30–39	6	29.11 ± 4.79	35.66 ± 4.38	1.24 ± 0.09	0.047	29.11 ± 3.08	1.01 ± 0.08	1.000
40–49	25	28.39 ± 5.13	34.97 ± 5.75	1.24 ± 0.11	<0.001	28.60 ± 4.07	1.02 ± 0.09	0.876
50–59	86	25.93 ± 5.04	31.05 ± 5.97	1.20 ± 0.14	<0.001	25.85 ± 4.22	1.01 ± 0.11	0.909
60–69	72	24.78 ± 5.24	29.70 ± 6.10	1.21 ± 0.13	<0.001	24.87 ± 4.35	1.02 ± 0.11	0.914
70–79	37	21.90 ± 4.96	25.13 ± 6.16	1.16 ± 0.18	0.017	21.59 ± 4.43	1.00 ± 0.14	0.782
>80	3	22.05 ± 3.30	24.98 ± 3.42	1.14 ± 0.03	0.431	21.49 ± 2.47	0.98 ± 0.04	0.857
Total	229	25.22 ± 5.43	30.14 ± 6.60	1.20 ± 0.14	<0.001	25.18 ± 4.70	1.01 ± 0.11	0.937
**Women**	**VO2max ± SD (mL/kg/min)**							
**Age**	**N**	**Direct**	**ACSM**	**Ratio**	***p*-Value**	**FRIEND**	**Ratio**	***p*-Value**
30–39	0							
40–49	3	24.15 ± 2.47	26.15 ± 1.04	1.09 ± 0.07	0.717	22.34 ± 0.78	0.93 ± 0.07	0.482
50–59	13	22.86 ± 4.78	27.10 ± 5.27	1.20 ± 0.14	0.075	23.01 ± 3.77	1.02 ± 0.12	0.991
60–69	27	21.09 ± 3.41	25.31 ± 3.78	1.21 ± 0.18	<0.001	21.72 ± 2.77	1.04 ± 0.14	0.532
70–79	18	18.01 ± 3.14	19.87 ± 5.33	1.09 ± 0.18	0.273	17.71 ± 3.96	0.98 ± 0.12	0.779
>80	3	13.30 ± 2.76	13.32 ± 5.83	0.96 ± 0.27	0.942	12.66 ± 4.62	0.93 ± 0.18	0.867
Total	64	20.36 ± 4.36	23.62 ± 5.85	1.16 ± 0.19	0.001	20.46 ± 4.33	1.01 ± 0.14	0.927

VO2max; (ml/kg/min), N; number, SD; standard deviation. ACSM; The American College of Sports Medicine, FRIEND; Fitness Registry and the Importance of Exercise National Database. The ratio is a comparison with the Direct method. *p*-values are statistical differences from Direct method.

**Table 4:** Equivalence analysis of the ACSM equation and the FRIEND equation.

Equation	ACSM	FRIEND
Mean bias error (MBE)	4.559	−0.008
CI	4.165–4.952	−0.320–0.304
Mean square error (MSE)	32.456	7.318
CI	28.489–36.433	5.881–8.755
Mean absolute percent error (MAPE)	20.963	8.937
CI	19.501–22.426	8.052–9.822
Lin’s concordance correlation coefficient (Lin’s CCC)	0.678	0.870
CI	0.642–0.711	0.841–0.895

CI; 95% confidence interval, MBE; mean value of [estimated VO2max − directly measured VO2max], MSE; mean value of [(estimated VO2max − directly measured VO2max)^2^], MAPE; mean value of [$\frac{\left|\mathrm{estimated}\;\mathrm{VO}2\max\;–\;\mathrm{directly}\;\mathrm{measured}\;\mathrm{VO}2\max\right|}{\mathrm{directly}\;\mathrm{measured}\;\mathrm{VO}2\max}$ × 100].

## Supplementary Materials

The following are available online at https://www.mdpi.com/2077-0383/9/6/1889/s1, Table S1: Equivalence analysis of the ACSM equation and the FRIEND equation according to medication. Table S2: Equivalence analysis of the ACSM equation and the FRIEND equation according to clinical presentation. Table S3: RER level comparison of CRF between direct measured CRF and estimated CRF by the ACSM equation and FRIEND equations.
