# Supplementary material for: Validation of FRIEND and ACSM Equations for Cardiorespiratory Fitness: Comparison to Direct Measurement in CAD Patients
Source: J Clin Med. 2020 Jun 17;9(6):1889. doi: 10.3390/jcm9061889 (PMC7356312; doi:10.3390/jcm9061889)
Supplement: Supplementary file 1 [file jcm-09-01889-s001.pdf]

Table S1. Equivalence analysis of the ACSM equation and the FRIEND equation according to medication.

Medication		Method	Mean ± SD (mL/kg/min)	MAPE	95% CI	Lin's CCC	95% CI
BB	Taking	Direct	25.09 ± 0.43				
		ACSM	29.90 ± 0.55	20.96	18.97 - 22.94	0.65	0.60 - 0.70
		FRIEND	25.00 ± 0.39	8.63	7.47 - 9.79	0.87	0.82 - 0.90
	Not taking	Direct	23.05 ± 0.48				
		ACSM	27.31 ± 0.60	20.97	18.78 - 23.16	0.69	0.63 - 0.74
		FRIEND	23.14 ± 0.43	9.30	7.93 - 10.68	0.86	0.82 - 0.90
CCB	Taking	Direct	23.85 ± 0.56				
		ACSM	28.43 ± 0.72	21.44	18.51 - 24.37	0.63	0.54 - 0.70
		FRIEND	23.95 ± 0.52	8.80	6.93 - 10.67	0.83	0.76 - 0.89
	Not taking	Direct	24.29 ± 0.40				
		ACSM	28.84 ± 0.50	20.76	19.07 - 22.45	0.70	0.66 - 0.73
		FRIEND	24.23 ± 0.36	8.99	8.00 - 9.99	0.88	0.85 - 0.91

SD; standard deviation, MAPE; mean absolute percent error, CI; confidence interval, Lin's CCC; Lin's concordance correlation coefficient, BB; beta blocker, CCB; calcium channel blocker, ACSM; The American College of Sports Medicine, FRIEND; Fitness Registry and the Importance of Exercise National Database.

Table S2. Equivalence analysis of the ACSM equation and the FRIEND equation according to clinical presentation.

Diagnosis	Method	Mean ± SD (mL/kg/min)	MAPE	95% CI	Lin's CCC	95% CI
Myocardial infarction	Direct	24.42 ± 0.52				
	ACSM	29.20 ± 0.67	20.37	17.97 - 22.76	0.66	0.61 - 0.71
	FRIEND	24.50 ± 0.48	8.13	6.88 - 9.38	0.89	0.85 - 0.92
Angina	Direct	24.01 ± 0.42				
	ACSM	28.44 ± 0.52	21.30	19.44 - 23.16	0.68	0.64 - 0.73
	FRIEND	23.95 ± 0.37	9.4	8.20 - 10.59	0.86	0.82 - 0.89

SD; standard deviation, MAPE; mean absolute percent error, CI; confidence interval, Lin's CCC; Lin's concordance correlation coefficient, ACSM; The American College of Sports Medicine, FRIEND; Fitness Registry and the Importance of Exercise National Database.

Table S3. RER level comparison of CRF between direct measured CRF and estimated CRF by the ACSM equation and FRIEND equations.

	VO2max ± SD		(mL/kg/min)		
	Direct	ACSM	p-value+	FRIEND	p-value+
RER ≥ 1.10	26.34 ± 5.19	32.39 ± 5.80	<0.001	26.78 ± 4.12	0.442
RER ≥ 1.0	24.93 ± 5.42	30.02 ± 6.48	<0.001	25.09 ± 4.65	0.736
RER < 1.0	20.62 ± 4.83	22.39 ± 5.84	0.109	19.62 ± 4.20	0.282
RER < 0.9	18.86 ± 4.53	19.65 ± 4.73	0.620	17.67 ± 3.35	0.390
p-value*	<0.001	<0.001		<0.001	

RER ≥ 1.0 includes RER ≥ 1.1 and RER < 1.0 also includes RER <0.9. p-value*; statistical difference between RER ≥ 1.0 and RER <1.0. p-value+; statistical difference between Direct methods and estimate methods. SD; standard deviation, RER; respiratory exchange ratio, ACSM; The American College of Sports Medicine, FRIEND; Fitness Registry and the Importance of Exercise National Database.
